# Gender Identification by Fingerprint Pattern and Salivary Blood Group Antigen Expression: A Forensic Approach

**DOI:** 10.7759/cureus.56324

**Published:** 2024-03-17

**Authors:** Sneha Masne Deshpande, Sheetal Choudhari, Pratibha Kavle, Amit Patil, Pallavi Kale

**Affiliations:** 1 Department of Oral Pathology and Microbiology, Bharati Vidyapeeth Dental College and Hospital, Navi Mumbai, IND; 2 Department of Oral and Maxillofacial Pathology, Yerala Medical Trust (YMT) Dental College, Mumbai, IND; 3 Department of Conservative Dentistry and Endodontics, Bharati Vidyapeeth Dental College and Hospital, Navi Mumbai, IND

**Keywords:** victim identification, forensic odontology, saliva, finger printing, blood grouping, dermatoglyphics

## Abstract

Introduction

Fingerprints found at the crime scene are important and valuable evidence, as they are unique to every individual. Determining the blood group from the blood samples obtained at the site of the crime helps in identifying a person. However, where blood stains are not available, saliva obtained at the crime site can be used to identify the victim. Since fingerprint patterns and blood groups are unique to every individual and remain unchanged throughout life, the correlation between dermatoglyphics and blood groups can be of use in victim identification.

Objectives

The present study is conducted with the objective of finding out if there is any association between the distribution of fingerprint patterns and blood groups and if this association is of use in gender identification.

Materials and method

Fingerprint patterns were determined in 200 (females: n = 152, males: n = 48) dental undergraduate students in the age range of 18 to 24 years. ABO blood grouping was done on saliva by using the absorption-elution method. To determine the accuracy of ABO blood group determination using saliva, it was correlated with the ABO blood grouping in blood.

Observations and result

The most common fingerprint pattern was found to be loops (87, 43.50%), followed by whorls (81, 40.50%) and arches (32, 16.00%). The most common blood group was B (68, 34%), followed by O (46, 23%) and A (42, 21%), and the least common was AB (12, 6%). A higher percentage of secretors in saliva was observed in females (130, 86%) than males (38, 79%). The correlation of gender with blood group and fingerprint pattern showed that in males, the most common blood group was B (20, 42%), and the most common fingerprint pattern was whorls (21, 44%). In females, the most common blood group was B (48, 32%), while the most common fingerprint pattern was loop (68, 45%).

Conclusion

Present study reports an association between blood group and dermatoglyphics, which may help in gender identification. Saliva can be used as a helpful tool in victim identification in cases where blood stains are not available.

## Introduction

Identification of humans is a prerequisite for personal, social, and legal reasons. Humans can be identified by various means, including physical and biological characteristics such as face recognition, friction ridge analysis, DNA finger-typing, age determination, post-mortem reports, blood groups, etc. Among these, dermatoglyphics is an important means of identification. Fingerprint patterns are genotypically determined and remain unchanged throughout the life of an individual [1]. Fingerprints assist in finding criminals at the crime site, biometric safety, etc. Each individual's fingerprints are unique, and there are subtle differences between the left and right thumbprints [2]. Fingerprints have immense potential to be used as an effective method of gender identification. The prints were determined using a magnifying glass, classified, and analyzed by the Cummins method of fingerprint identification into whorls, loops, and arches.

The blood group remains unchanged from birth to death and, therefore, can serve as the best source of evidence. Apart from blood, blood group antigens are also secreted in various body secretions such as saliva, tears, urine, semen, sweat, vaginal secretions, etc. Saliva has many perquisites and is considered beneficial over blood because, unlike blood, its collection is non-invasive, easy, and safer, and it has a low risk of contamination, especially from transmitted diseases such as hepatitis [3,4]. In cases where blood stains cannot be found at the crime site, saliva can be effectively utilized for blood grouping. Wet or dry saliva obtained from articles contaminated with it at the crime site can be used for blood grouping. As the fingerprint pattern and blood group antigen are unique and remain unchanged throughout life, finding an association between the two can be of use in victim identification.

## Materials and methods

The present study was conducted with the voluntary participation of 200 undergraduate dental students in the age range of 18-24 years at a government-aided private college after obtaining approval from the Institutional Ethics Committee. Demographic details of the participants, like name, age, and sex, were noted.

**Figure 1:**
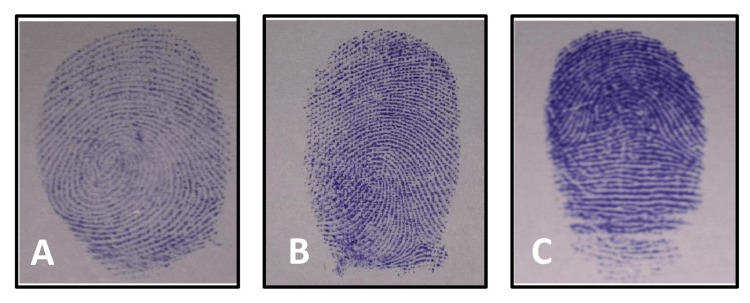
Different fingers print patterns loops (A), arches (B), and whorls (C).

Recording the fingerprints: Participants with normal thumbs were included in the study. Students with permanent scars on their fingers or thumbs, with any hand deformities due to injury, birth defect, or disease, or those who have worn fingerprints, extra-webbed or bandaged fingers, or hypersensitivity to endorsing ink were excluded from the study. Before taking fingerprints, the participants were asked to clean their hands with soap to remove any dirt and grease to obtain clean and legible fingerprints. The subjects were asked to give imprints of their left thumb by using the ink method as described by Cummins and Midlo [5]. A magnifying glass was used to identify and study the fingerprint patterns. They were identified as loops, whorls, and arcs (Figure 1) based on the appearance of ridge lines according to Henry's system of classification. 

**Figure 2:**
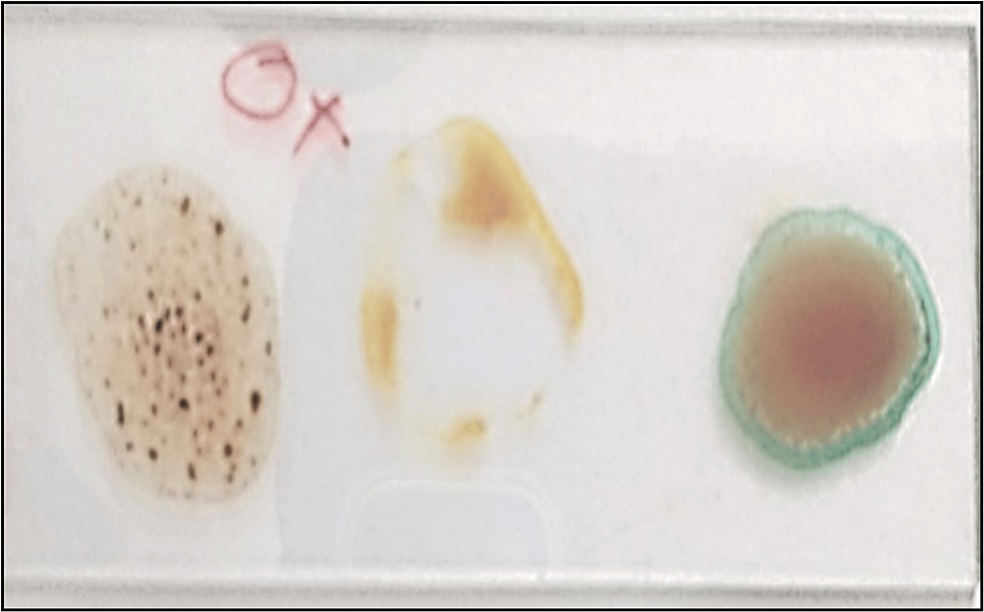
Photograph showing the Ag-Ab reaction.

Blood grouping: ABO blood grouping was carried out on saliva by using the absorption-elution method [6], which was then correlated with that of blood. Blood groups were assessed on blood by the slide agglutination method [7] (Figure 2). The perceived blood group was considered a standard against which the salivary blood group was compared. 

**Figure 3:**
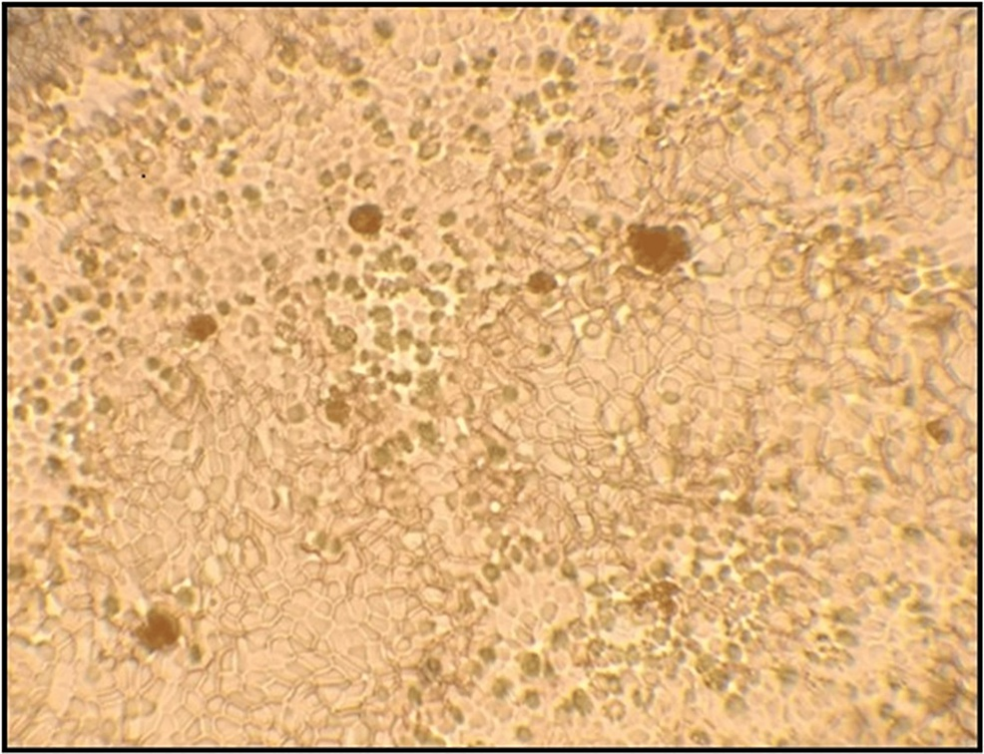
Photomicrographs showing masses of agglutinates. Photomicrograph showing agglutination of red blood corpuscles (RBCs) under magnification of the 40x objective of the microscope. No stain is used, and the slide is directly observed under a microscope for agglutination.

Absorption-elution method: This method is highly sensitive, and even a small amount of stain may be sufficient to get the best results [7]. ABO blood group estimation from saliva was done by the absorption elution method. 1.5 ml of unstimulated saliva was taken in an Eppendorf tube placed in a boiling water bath for 10 minutes. After discarding the sediment, the supernatant was taken into two test tubes and labeled A and B, to which anti-sera A and B were added, respectively. The sample was thoroughly shaken and allowed to incubate for 5 hours to obtain an antigen-antibody reaction. Following incubation, the excess antibody was removed by cold saline washes, which were repeated five times. Then, the sample was heated in a hot water bath for 560°C to break bonds between Ag and Ab. A single drop of freshly prepared RBCs from a known blood group was added to the tube and shaken, which was again incubated for 15 minutes at 370°C. Both the test tubes were then centrifuged for 1 minute at 2000 rpm. The presence of agglutination was noted microscopically (Figure 3), and the presence of agglutination was considered a positive result.

The collected data was subjected to statistical analysis using frequency distribution and the Chi-square test with the help of IBM Corp. Released 2011. IBM SPSS Statistics for Windows, Version 20.0. Armonk, NY: IBM Corp. A p-value of < 0.05 was considered statistically significant for the association of variables.

## Results

The fingerprint pattern and blood grouping of 200 dental students (152 females and 48 males) were studied. The dominant pattern among the entire study group was found to be loops (87, 43.50%), followed by whorls (81, 40.50%) and arches (32, 16.00%) (Table 1). ﻿

**Table 1:** Showing distribution of fingerprint pattern in males and females.

Fingerprint pattern in gender			Gender		Total
			Male	Female	
Fingerprint pattern	Loops	(n) Count	n=19	n=68	n=87
		% Within Gender	39.6%	44.7%	43.5%
		% Of Total	9.5%	34.0%	43.5%
	Whorls	(n) Count	n=21	n=60	n=81
		% Within Gender	43.8%	39.5%	40.5%
		% Of Total	10.5%	30.0%	40.5%
	Arches	(n) Count	n=8	n=24	n=32
		% Within Gender	16.7%	15.8%	16.0%
		% Of Total	4.0%	12.0%	16.0%
Total		(n) Count	n=48	n=152	n=200
		% Within Gender	100.0%	100.0%	100.0%
		% Of Total	24.0%	76.0%	100.0%

In males, the most common fingerprint pattern was whorls (21, 43.8%), followed by loops (19, 39.6%) and arches (8, 16.7%). Females showed the most common pattern as loops (68, 44.7%), followed by whorls (60, 39.5%) and arches (24, 15.8%). The most common blood group through saliva was found to be B (68, 34%), followed by O (46, 23%), A (42, 21%), and AB (12, 6%) (Table 2). 

**Table 2:** Showing expression of saliva blood group antigen in males and females.

Saliva Blood Group Antigen			Gender		Total
			Male	Female	
Saliva Blood Group Antigen	A	(n) Count	n=6	n=36	n=42
		% within Gender	12.5%	23.7%	21.0%
		% of Total	3.0%	18.0%	21.0%
	B	(n) Count	n=20	n=48	n=68
		% within Gender	41.7%	31.6%	34.0%
		% of Total	10.0%	24.0%	34.0%
	AB	(n) Count	n=2	n=10	n=12
		% within Gender	4.2%	6.6%	6.0%
		% of Total	1.0%	5.0%	6.0%
	O	(n) Count	n=10	n=36	n=46
		% within Gender	20.8%	23.7%	23.0%
		% of Total	5.0%	18.0%	23.0%
	NO EXPRESSION	(n) Count	n=10	n=22	n=32
		% within Gender	20.8%	14.5%	16.0%
		% of Total	5.0%	11.0%	16.0%
Total		(n) Count	n=48	n=152	n=200
		% within Gender	100.0%	100.0%	100.0%
		% of Total	24.0%	76.0%	100.0%

**Figure 4:**
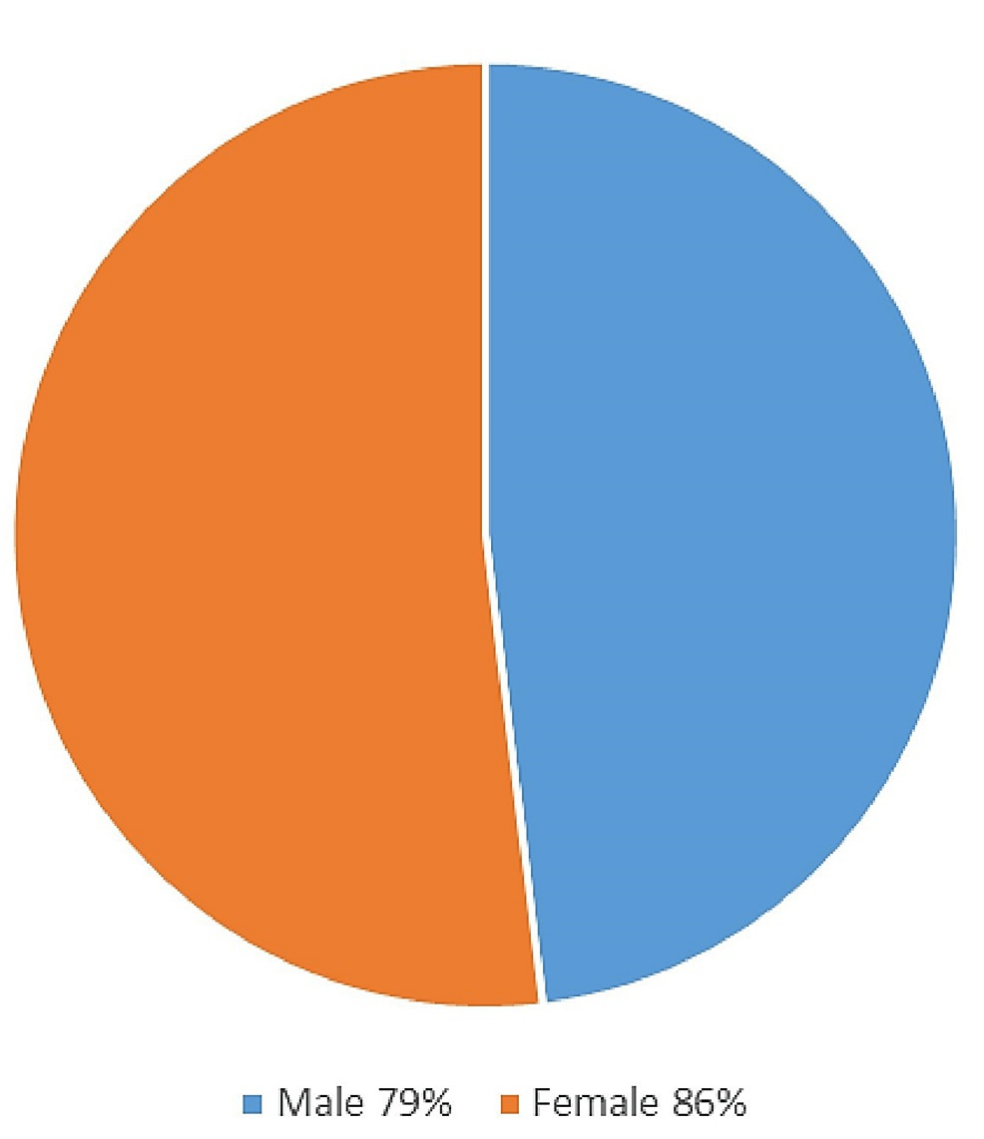
Percentage of salivary secretors in males and females.

When the ABO blood grouping using saliva was correlated with that of blood, a higher percentage of secretors in saliva was observed in females (130, 86%) than males (38, 79%) (Figure 4).

Table 3 shows the distribution of fingerprint patterns among the A, B, O, and AB blood groups on saliva in males and females. In males, the most common fingerprint pattern was whorls (21, 44%), and the blood group was B (20, 42%). In females, the most common fingerprint pattern was loop (68, 45%), and the most common blood group was B (48, 32%). 

**Table 3:** Distribution of blood group (A, B, O, AB) and fingerprint pattern in males and females.

Gender		Male	Female
Saliva Blood Group Antigen	A	n= 6 (12.5%)	n=36 (23.7%)
	B	n=20 (41.7%)	n=48 (31.6%)
	AB	(n=2) 4.2%	(n=10) 6.6%
	O	(n=10) 20.8%	(n=36) 23.7%
	No Expression	(n=10) 20.8%	(n=22) 14.5%
Fingerprint pattern	Loops	(n=19) 39.6%	(n=68) 44.7%
	whorls	(n=21) 43.8%	(n=60) 39.5%
	Arches	(n=8) 16.7%	(n= 24) 15.8%

## Discussion

Fingerprints are unique and considered the gold standard for revealing an individual's identity [8]. Blood is one of the prime investigative methods in forensic medicine [9]. Blood grouping helps in knowing a person’s identity in medicolegal cases. Knowing the association between these two unique identification sources can be effective in the forensic sciences for victim identification. Expression of blood group antigens is usually tested on blood. However, saliva can be used as a non-invasive, convenient, and safe tool for blood grouping. The present study finds an association between fingerprint pattern and salivary blood group antigen in identification.

In this study, the most common fingerprint pattern among the entire population was found to be loops (87, 43.50%), followed by whorls (81, 40.50%), and the least common was arches (32, 16.00%). These findings are similar to those reported by Fayrouz NE et al. in 2012 [10], Shrestha I et al. in 2019 [11], and Anyanwu LC et al. in 2020. [12] The present study reports whorls (21, 43.8%) as the most common fingerprint pattern in males, followed by loops (19, 39.6%) and arches (8, 16.7%), while in females, the most common pattern was loops (68, 44.7%), followed by whorls (60, 39.5%), and the least common pattern was arches (24, 15.5%). These results are in accordance with the studies by Nithin MD et al. [13] and Patil A et al. [14]. The results of the present study suggest that saliva can be an effective tool for blood group determination. This study shows that, overall, 84% of salivary secretors are among the study population. A higher percentage of secretors in saliva was observed in females (130, 86%) than males (38, 79%). Studies have shown that Indian people are more likely to secrete blood group antigens in their saliva than other races. Females are found to be better secretors of blood group antigens than males. This is in accordance with the studies by Motghare P et al. (2011) [15] and Bokhedher R et al. (2020) [16]. Saliva can serve as a less invasive and easy way of identifying blood groups in comparison to blood. It can be useful in forensic medicine where blood stains are unavailable. However, further research is required to increase the sensitivity of saliva for blood group identification.

In this study, an association between the distribution of fingerprint patterns and salivary blood groups was explored for gender identification. In males, the most common fingerprint pattern was whorls (21, 43.8%), and the blood group was B (20, 41.7%). This study shows that for blood group A (6, 12.5%) in males, the most common fingerprint pattern was loop (19, 39.6%), and in females the most common fingerprint pattern was loop (20, 10%), followed by whorls (16, 8%). Similarly, for blood group B (20, 41.7%) in males, the most common fingerprint pattern was whorls (21, 10%), and in females, the most common fingerprint pattern was loop (48, 24%); likewise, for blood group O, in males, the most common fingerprint pattern was a loop (9, 4.5%), and in females, the most common fingerprint pattern was whorls (36, 18%) (Table 4).

**Table 4:** Percentage of gender expression with different blood groups.

Saliva Blood Group Antigen	Gender	Fingerprint Pattern	%
A	M	Loop	3.00%
A	M	Whorl & Arch	0.00%
B	M	Whorls	10.00%
B	M	Loop & Arch	0.00%
AB	M	Loop	1.00%
AB	M	Whorl & Arch	0.00%
O	M	Whorl	0.50%
O	M	Loop	4.50%
O	M	Arch	0.00%
No Expression	M	Loop	1.00%
No Expression	M	Arch	4.00%
No Expression	M	Whorl	0.00%
A	F	Loop	10.00%
A	F	Whorl	8.00%
A	F	Arch	0.00%
B	F	Loops	24.00%
B	F	Whorl & Arch	0.00%
AB	F	Whorl	4.00%
AB	F	Arch	1.00%
AB	F	Loop	0.00%
O	F	Whorl	18.00%
O	F	Arch & Loop	0.00%
No Expression	F	Arch	11.00%
No Expression	F	Loop & Whorl	0.00%

Based on the observation, it can be inferred that if an individual has blood group B and the fingerprint pattern is whorls, then the chance of gender expression being male is 10%. Similarly, if the fingerprint pattern is a loop, then the chance of gender expression being female is 24%. Likewise, if an individual has blood group O and the fingerprint pattern is loop, then the chance of gender expression being male is 4.5%. Likewise, if the fingerprint pattern is whorl, then the chance of gender expression being female is 18%. To obtain more sensitive and accurate results, it is recommended to include a larger sample size in the study.

When analyzing blood evidence at a crime scene, forensic investigators encounter various challenges. While identifying blood itself is relatively straightforward, detecting specific antigens and other substances in secretions and tissues can be more complex. However, the examination of secretions and tissues can indeed provide valuable insights, especially when blood evidence is absent or limited.

The limited availability of saliva samples at crime scenes can pose significant challenges to forensic analysis. When the quantity of the sample is insufficient, it becomes difficult or even impossible to extract detailed information from it. The consumption or destruction of samples during testing not only limits further analysis or retesting but also undermines the ability to replicate findings, validate results, or explore additional hypotheses. This limitation can have far-reaching implications, as it affects the reliability and reproducibility of forensic evidence.

The limitations of the study regarding the definitive role of the association of salivary blood group and fingerprint in forensic dental sciences suggest that further research is needed to fully understand their significance. This indicates that, while the study may have provided some insights, there are still unanswered questions and complexities that need to be addressed.

Additionally, the mention of advanced molecular techniques to improve the sensitivity and specificity of saliva in forensic analysis highlights a potential avenue for future research. This suggests that current methods may not be sufficiently sensitive or specific and that advancements in molecular techniques could enhance the accuracy and reliability of saliva-based forensic investigations.

Overall, the limitations identified in the study point towards opportunities for further research and the development of more sophisticated techniques in the forensic dental sciences. These advancements could lead to more accurate and reliable forensic analyses, ultimately improving the effectiveness of criminal investigations.

Previous studies on the association between fingerprint patterns and blood groups have primarily studied blood samples to detect the presence of blood group antigens. However, the present study reports the utility of saliva for blood group identification and the association between salivary blood groups and fingerprint patterns for gender identification. Further research is needed to determine the consistency of an association between fingerprint patterns and blood groups across different regions and populations.

## Conclusions

The present research paves the path for different sources, such as salivary blood grouping and an association between salivary blood groups and dermatoglyphics, for gender identification in forensic medicine. The potential use of saliva in forensic medicine should be explored more in view of its sustainability and availability at the crime site.
